# Supplementary material for: In vivo reprogramming drives Kras-induced cancer development
Source: Nat Commun. 2018 May 25;9:2081. doi: 10.1038/s41467-018-04449-5 (PMC5970190; doi:10.1038/s41467-018-04449-5)
Supplement: Supplementary file 1 — Supplementary Information [file 41467_2018_4449_MOESM1_ESM.pdf]

**_In vivo_ reprogramming drives _Kras_-induced cancer development**

Shibata et al.

## Inventory of Supplemental Information

**Supplemental Figures (Figure S1-Figure S9)**

**Legends to Supplemental Figures**

**Supplemental Table 1-Table 7**

# Supplementary Figure 1

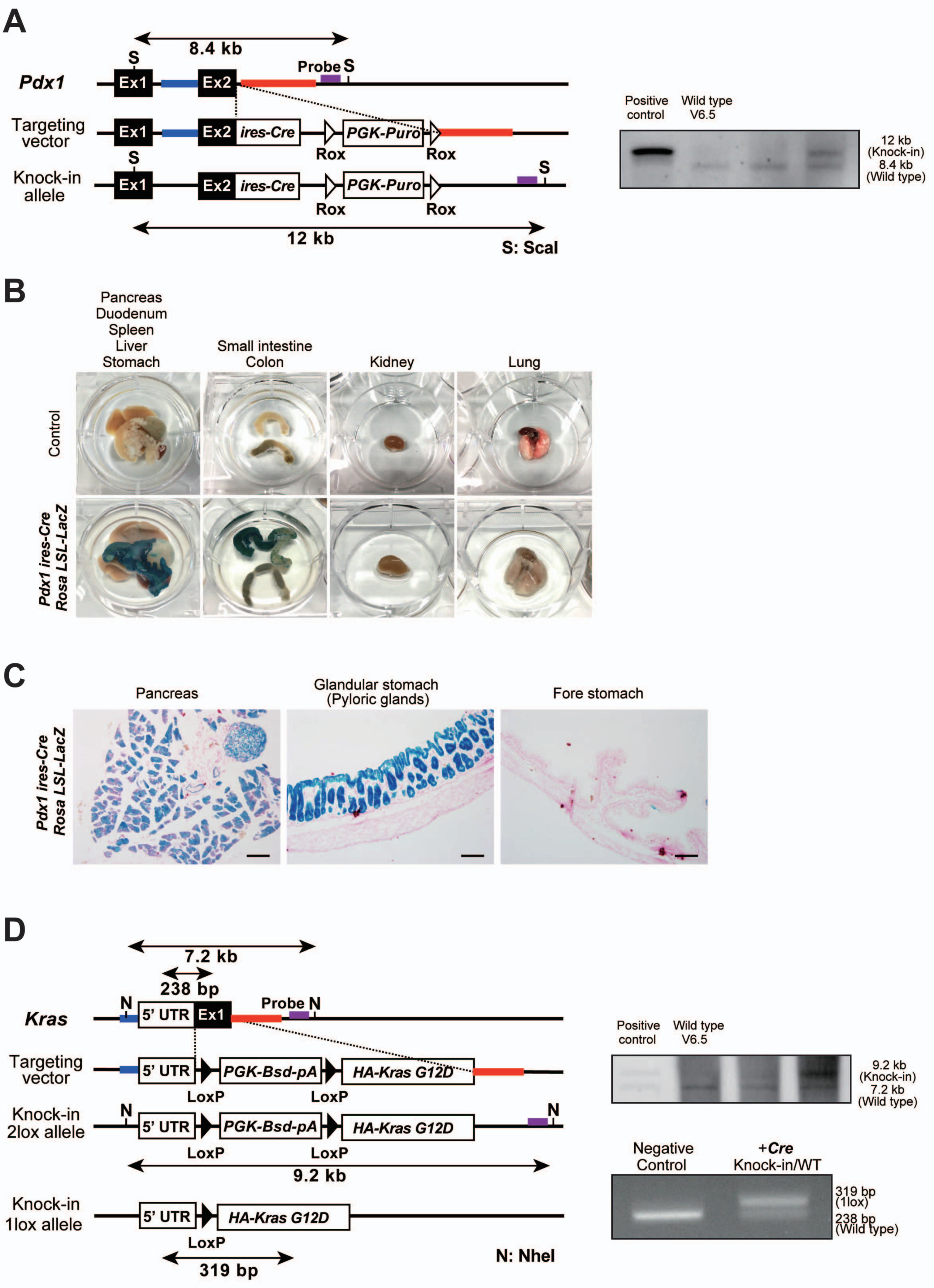

**Supplementary Figure 1: *Kras/p53* compound mutations are insufficient for PDAC development.**

(A) A schematic illustration of homologous recombination with a targeting vector containing *ires-Cre* at endogenous *Pdx1* locus. Southern blotting indicates successful recombination in ESCs.

(B) Whole mount LacZ staining of various organs of *Pdx1 ires-Cre, Rosa LSL-LacZ* mice.

(C) LacZ staining on the histological sections of the pancreas and stomach of *Pdx1 ires-Cre, Rosa LSL-LacZ* mice. Recombination is observed in most pancreatic cells and pyloric glands in the stomach. Scale bars, 100 μm.

(D) A schematic illustration of homologous recombination with a targeting vector containing *LSL-HA tag-Kras G12D* allele at endogenous *Kras* locus. Southern blotting indicates successful recombination in ES cells. 1*LoxP* PCR confirms excision of *LSL* cassette of *LSL-HA tag-Kras G12D* allele in the +/KI pancreas with *Pdx1 ires-Cre* allele.

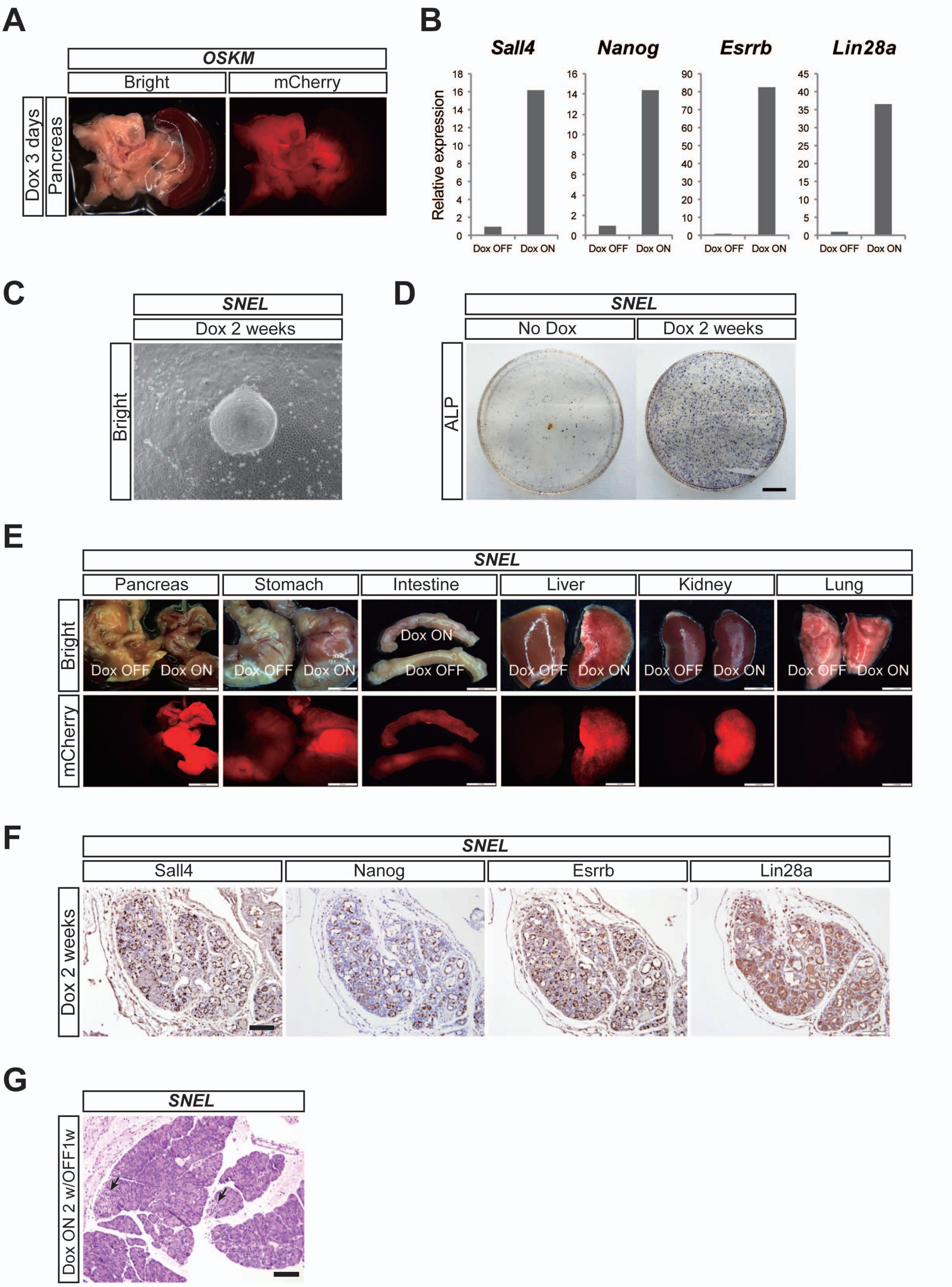
A
OSKM
Bright
mCherry
Dox 3 days
Pancreas
B
Sall4
Nanog
Esrrb
Lin28a
Relative expression
Dox OFF
Dox ON
C
SNEL
Dox 2 weeks
Bright
D
SNEL
No Dox
Dox 2 weeks
ALP
E
SNEL
Pancreas
Stomach
Intestine
Liver
Kidney
Lung
Bright
mCherry
Dox OFF
Dox ON
F
SNEL
Sall4
Nanog
Esrrb
Lin28a
Dox 2 weeks
G
SNEL
Dox ON 2 w/OFF1w

**Supplementary Figure 2; Transient expression of reprogramming factors induces reversible acinar to ductal metaplasia.**

(A) mCherry expression in the pancreas of Dox-treated *C-OSKM* mice.

(B) A RT-qPCR analysis of *Sall4, Nanog, Esrrb,* and *Lin28a* transgenes in KH2-*SNEL* MEFs after Dox-exposure for 3 days. Data are presented as the mean of technical triplicates. The mean expression level of Dox OFF samples was set to 1.

(C) iPSC-like colony emerged after Dox treatment for 2 weeks in KH2-*SNEL* MEFs.

(D) Alkaline phosphatase staining on KH2-*SNEL* MEFs after Dox exposure for 2 weeks. Scale bar, 1 cm.

(E) mCherry fluorescence is detectable in the pancreas, stomach, intestine, liver, kidney and lung of KH2-*SNEL* chimeric mice given Dox for 3 days. Scale bars, 10 mm.

(F) Immunostaining for Sall4, Nanog, Esrrb, and Lin28a in the pancreas of Dox-treated KH2-*SNEL* chimeric mice. Scale bar, 100 μm.

(G) Although a small number of acini exhibit slight dilatation (arrows), ADM lesions are hardly detectable after Dox withdrawal for 1 week in the pancreas of Dox-treated KH2-*SNEL* chimeric mice. Scale bars, 100 μm.

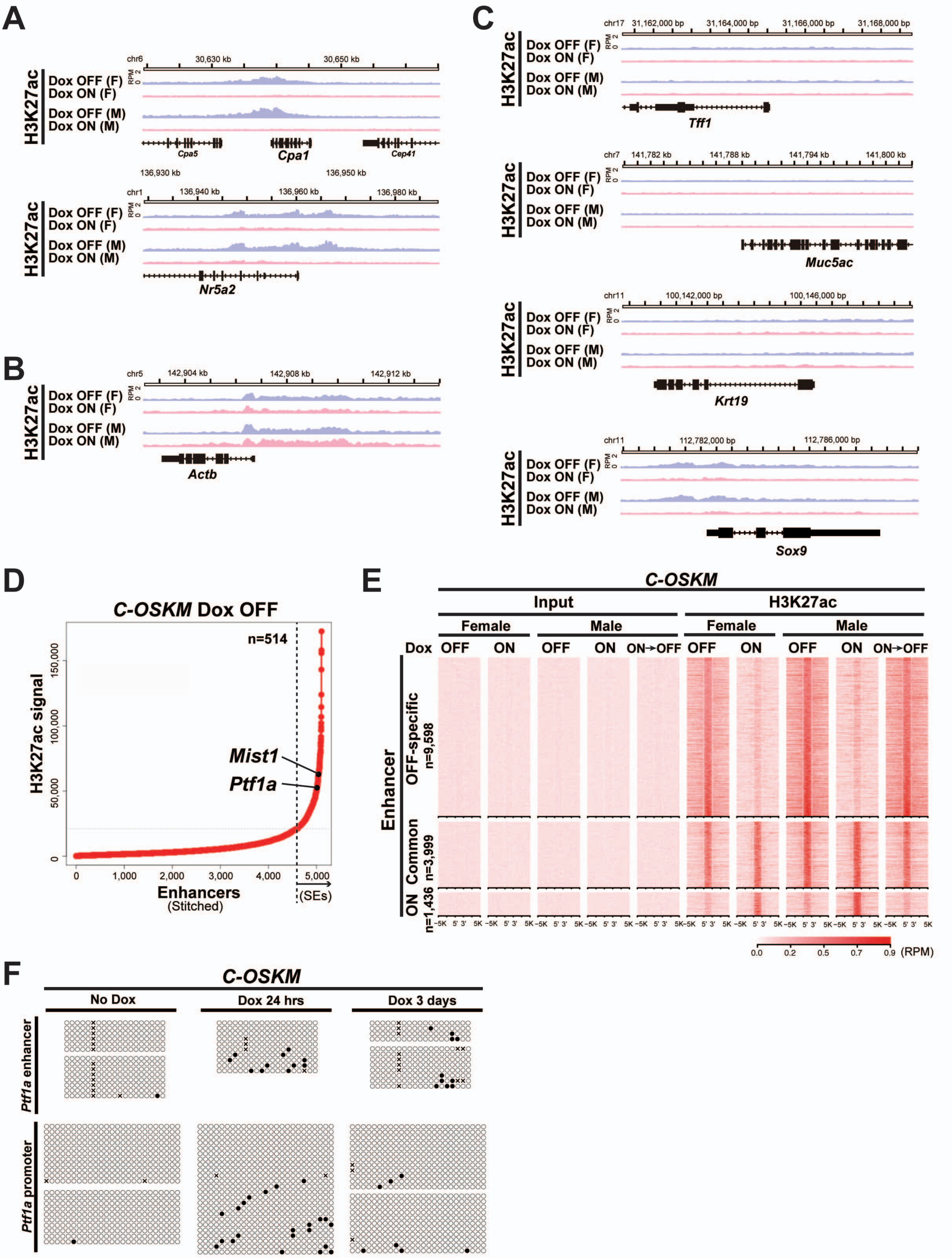
A
H3K27ac
Dox OFF (F)
Dox ON (F)
Dox OFF (M)
Dox ON (M)
chr6
30,630 kb
30,650 kb
Cpa5
Cpa1
Cep41
136,930 kb
136,950 kb
chr1
136,940 kb
136,960 kb
136,980 kb
Nr5a2
B
chr5
142,904 kb
142,908 kb
142,912 kb
Actb
C
chr17
31,162,000 bp
31,164,000 bp
31,166,000 bp
31,168,000 bp
Tff1
chr7
141,782 kb
141,788 kb
141,794 kb
141,800 kb
Muc5ac
chr11
100,142,000 bp
100,146,000 bp
Krt19
chr11
112,782,000 bp
112,786,000 bp
Sox9
D
C-OSKM Dox OFF
n=514
H3K27ac signal
Mist1
Ptf1a
Enhancers
(Stitched)
(SEs)
E
C-OSKM
Input
H3K27ac
Female
Male
Dox
OFF
ON
ON→OFF
Enhancer
OFF-specific n=9,598
Common n=3,999
ON n=1,436
(RPM)
F
C-OSKM
No Dox
Dox 24 hrs
Dox 3 days
Ptf1a enhancer
Ptf1a promoter

**Supplementary Figure 3: Repression of acinar cell enhancers by *in vivo* reprogramming.**

(A) ChIP-seq analysis for H3K27ac in the pancreas of *C-OSKM* mice. H3K27ac deposition is reduced at acinar cell-related genes, *Cpa1* and *Nr5a2*.

(B) H3K27ac deposition is not altered at a control gene (*Actb*).

(C) H3K27ac deposition is not increased at *Sox9* or ADM-related upregulated genes (*Tff1*, *Muc5ac* and *Krt19*).

(D) Super-enhancers (SEs) identified in the Dox OFF control pancreas. Note that SEs are observed at acinar cell-related transcription factors *Ptf1a* and *Mist1*.

(E) Enhancers in the pancreas of *C-OSKM* mice. Note that Dox OFF-specific enhancers show substantial reduction in H3K27ac deposition after *OSKM* induction.

(F) DNA methylation at *Ptf1a* promoter and enhancer. *Ptf1a* promoter and enhancer remain unmethylated after Dox treatment for 3 days.

Supplementary Figure 4

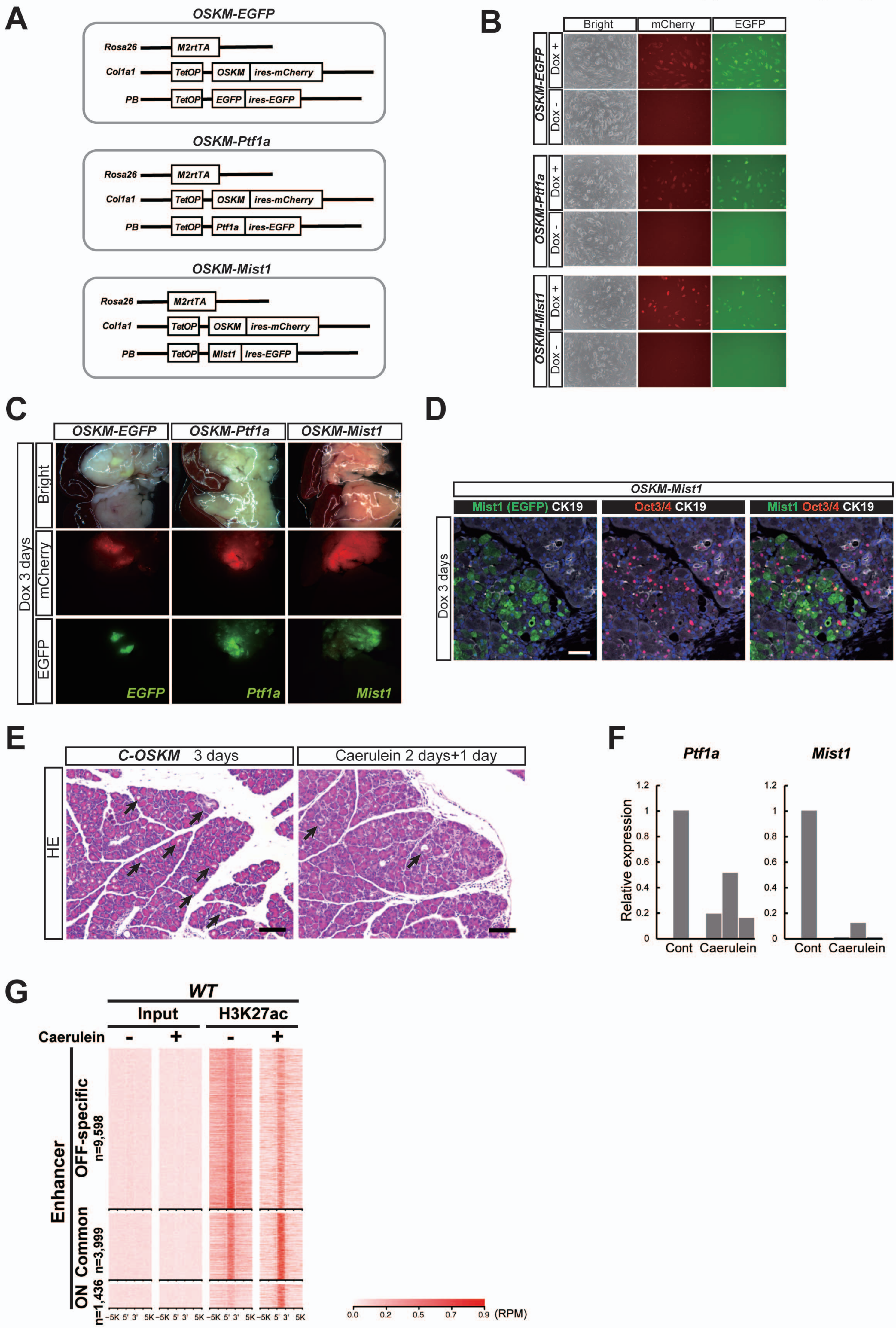

**Supplementary Figure 4: Forced expression of acinar cell-related transcription factors attenuates *OSKM*-induced ADM formation.**

(A) A schematic illustration of the genetic construct of *Rosa-M2rtTA, Col1a1::tetO-OSKM-ires-mCherry, PB-EGFP/Ptf1a/Mist1-ires EGFP* (*OSKM-EGFP, OSKM-Ptf1a* and *OSKM-Mist1*) ES cells.

(B) Simultaneous expression of mCherry and EGFP is observed in *OSKM-EGFP, OSKM-Ptf1a* and *OSKM-Mist1* ESCs after Dox exposure.

(C) Dox-treated *OSKM-EGFP, OSKM-Ptf1a* and *OSKM-Mist1* chimeric mice showing expression of mCherry and EGFP in the pancreas.

(D) Heterogeneous expression of EGFP (*Mist1*) is observed in Oct3/4-expressing pancreatic cells of *OSKM-Mist1* chimeric mice. Note that CK19 expression is attenuated in the EGFP (*Mist1*)-expressing acinar area.

(E) The pancreatic tissue with *OSKM* induction phenocopies caerulein-induced ADM lesions. Arrows indicate ADM lesions. Scale bars, 100 μm.

(F) A qRT-PCR for acinar cell-related genes after caerulein treatment in the pancreas. Data are presented as the mean of technical triplicates. The expression level of wild type sample was set to 1.

(G) Enhancers in caerulein-treated pancreas. Note that altered H3K27ac deposition in *OSKM*-induced pancreas are similarly altered by caerulein treatment.

# Supplementary Figure 5

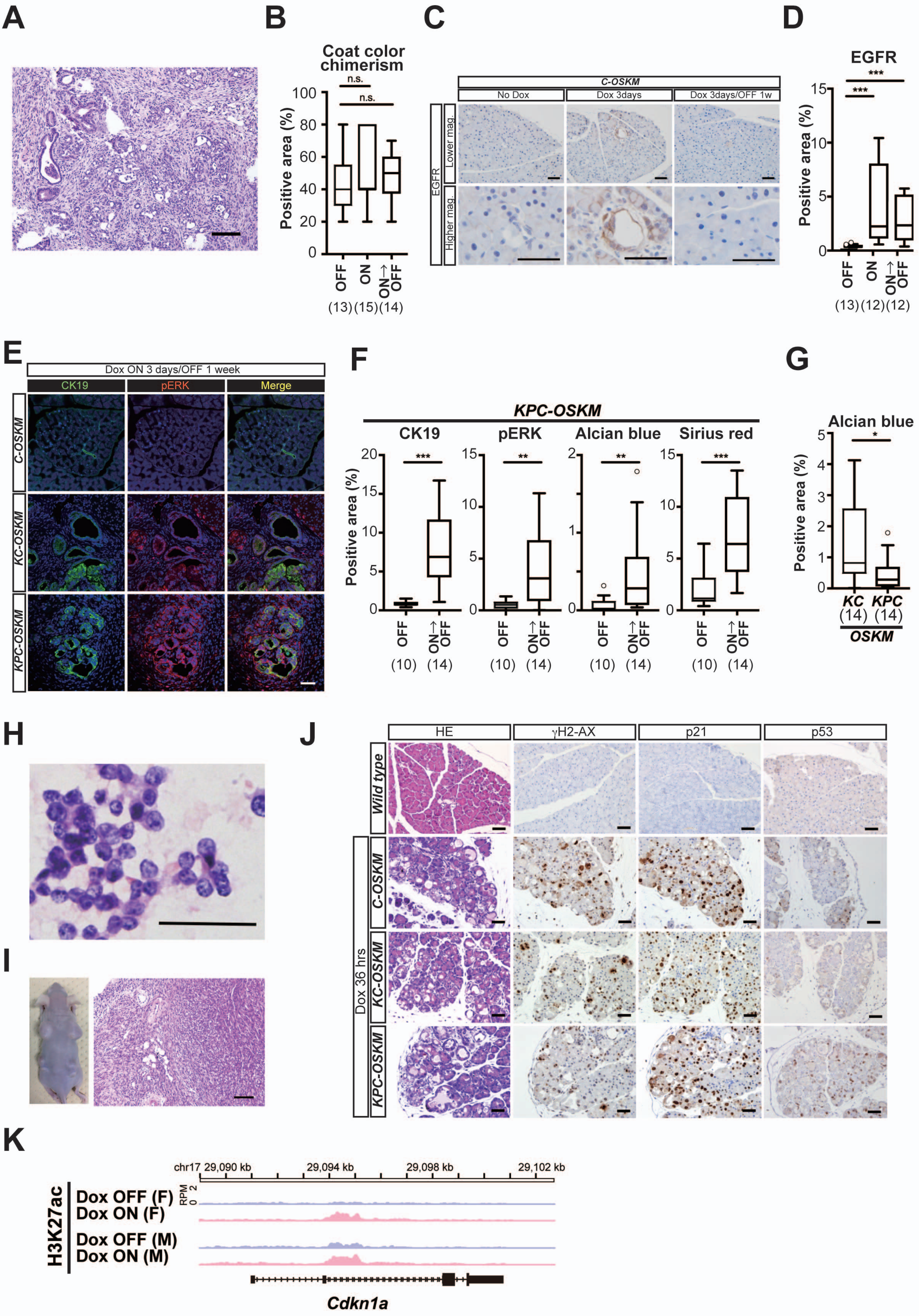

**Supplementary Figure 5; *In vivo* reprogramming in *Kras*/ *p53* compound mice causes rapid PDAC development.**

(A) Representative histological image of the pancreas of *KC-OSKM* chimeric mice. Scale bars, 100 μm.

(B) Coat color chimerism of *KC-OSKM* chimeric mice used for quantification of various stainings. A box-and-whisker plot of the chimerism. Solid lines in each box indicate the median. Bottom and top of the box are lower and upper quartiles, respectively. Whiskers extend to ±1.5 interquartile range (IQR). Numbers in parentheses indicate the number of mice examined. n.s.; not significant.

(C) Immunostaining for EGFR in the pancreas of *C-OSKM* mice. Modest EGFR expression is induced by Dox treatment. In contrast to *KC-OSKM* chimeric mice, EGFR expression is hardly detectable after Dox withdrawal for 1 week in *C-OSKM* chimeric mice. Scale bars represent 10 μm.

(D) Quantification of EGFR-positive area in the pancreas of *KC-OSKM* chimeric mice. Note that sustained EGFR expression is observed in *KC-OSKM* chimeric mice. A box-and-whisker plot of the EGFR positive area. Solid lines in each box indicate the median. Bottom and top of the box are lower and upper quartiles, respectively. Whiskers extend to ±1.5 interquartile range (IQR). Numbers in parentheses indicate the number of mice examined. ***$p < 0.001$.

(E) Immunostaining for CK19 and pERK in *KC-OSKM* and *KPC-OSKM* chimeric mice given Dox for 3 days, followed by Dox withdrawal for 1 week. Scale bar, 50 μm.

(F) Quantification of CK19-, pERK-, Alcian blue-, and Sirius red-positive areas in the pancreas of *KPC-OSKM* chimeric mice. A box-and-whisker plot of the positive area. Solid lines in each box indicate the median. Bottom and top of the box are lower and upper quartiles, respectively. Whiskers extend to ±1.5 interquartile range (IQR). Numbers in parentheses indicate the number of mice examined. **$p < 0.01$, ***$p < 0.001$.

(G) Quantification of Alcian blue-positive area for the pancreas of *KC-OSKM* and *KPC-OSKM* chimeric mice. A box-and-whisker plot of the positive area. Solid lines in each box indicate the median. Bottom and top of the box are lower and upper

quartiles, respectively. Whiskers extend to ±1.5 interquartile range (IQR). Numbers in parentheses indicate the number of mice examined. *$p < 0.05$.

(H) Dox-treated *KPC-OSKM* mice often develop malignant ascites. Scale bar, 50 μm.

(I) Cell lines derived from pancreatic tumors in Dox-treated *KPC-OSKM* mice develop secondary tumors in the subcutaneous tissue of immuno-deficient mice. Scale bar, 100 μm.

(J) Representative images for histology, γH2-AX, p21, p53 immunostaining of the pancreas in *C-OSKM, KC-OSKM,* and *KPC-OSKM* mice given Dox for 36 hours. Scale bars, 50 μm.

(K) ChIP-seq analysis for H3K27ac in the pancreas of *C-OSKM* mice. Increased H3K27ac deposition is observed at *Cdkn1a* (p21).

# Supplementary Figure 6

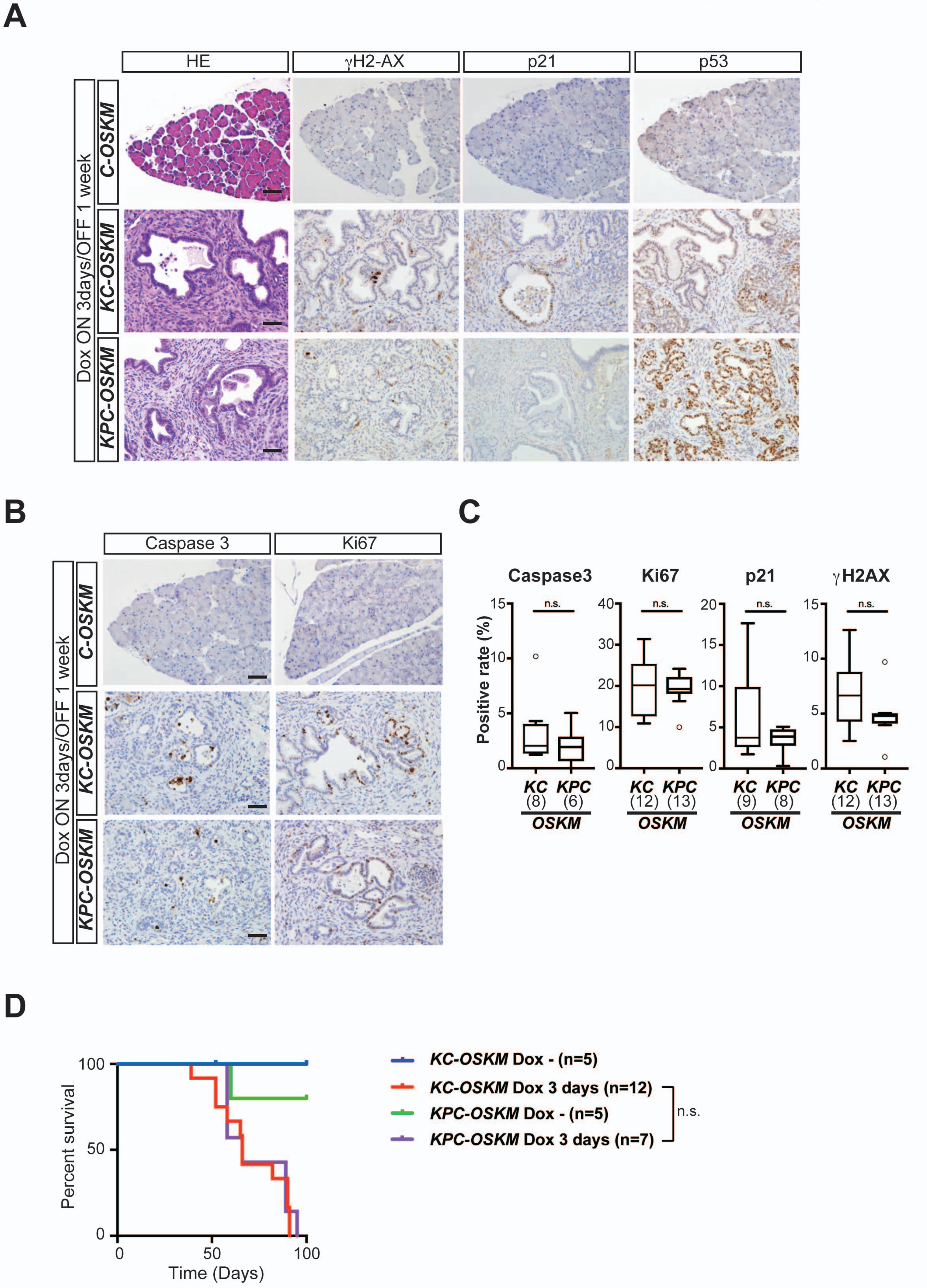

**Supplementary Figure 6: *In vivo* reprogramming in *Kras/p53* compound mice causes rapid PDAC development.**

(A) Representative images for histology, γH2-AX, p21, and p53 immunostaining in the pancreas of *C-OSKM, KC-OSKM, KPC-OSKM* mice given Dox for 3 days followed by Dox withdrawal for 1 week. Scale bars, 50 μm.

(B) Immunostaining for Caspase 3 and Ki67 in the pancreas of *C-OSKM, KC-OSKM, KPC-OSKM* mice given Dox for 3 days followed by Dox withdrawal for 1 week. Scale bars, 50 μm.

(C) Quantification of Caspase 3-, Ki67-, p21- and γH2AX- positive ratio in the pancreas of *KC-OSKM* and *KPC-OSKM* chimeric mice given Dox for 3 days followed by Dox withdrawal for 1 week. A box-and-whisker plot of the positive area. Solid lines in each box indicate the median. Bottom and top of the box are lower and upper quartiles, respectively. Whiskers extend to ±1.5 interquartile range (IQR). Numbers in parentheses indicate the number of mice examined. n.s.; not significant.

(D) Survival curve of *KC-OSKM* and *KPC-OSKM* mice with/without transient Dox treatment for 3 days starting at 4-weeks of age. Kaplan-Meier analysis was performed. n.s.; not significant.

## Supplementary Figure 7

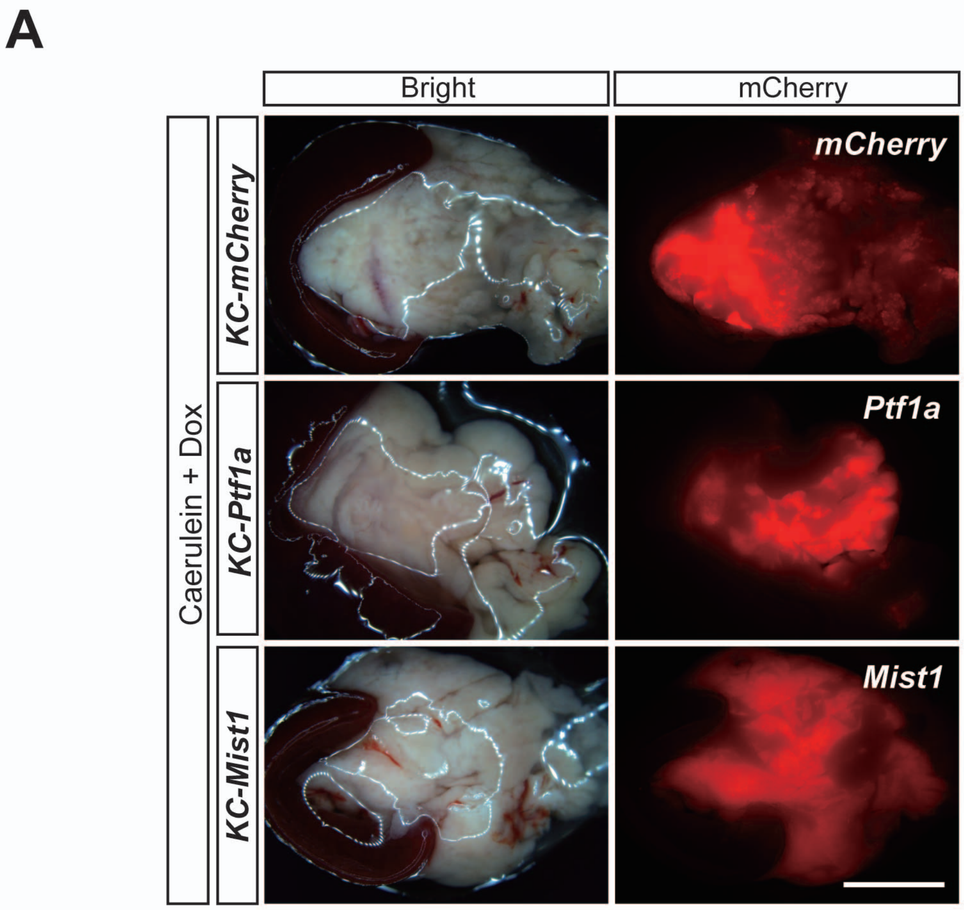

**Supplementary Figure 7: Forced expression of acinar cell-related transcription factor attenuates pancreatitis-induced activation of ERK signaling and ADM formation in *Kras*-mutated pancreatic cells.**

(A) *KC-mCherry*, *KC-Ptf1a* and *KC-Mist1* chimeric mice were given Dox starting at 24 hours before caerulein treatment and sacrificed after 7 days of caerulein/Dox treatment. Scale bars, 1 cm.

# Supplementary Figure 8

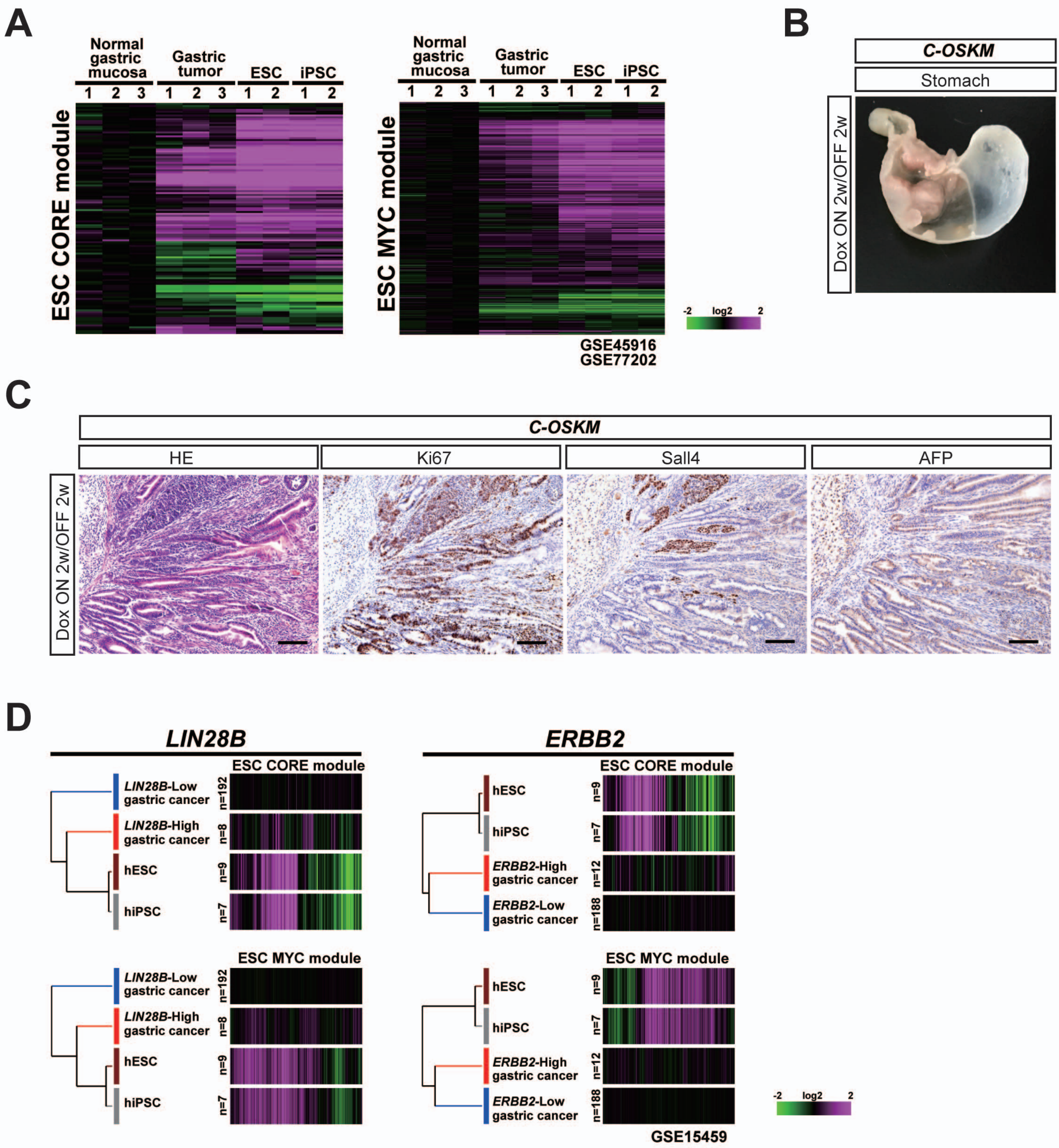

**Supplementary Figure 8: Prolonged expression of reprogramming factors induces poorly- differentiated cancers in *KC-OSKM* chimeric mice.**

(A) Partial activation of ESC-Core and ESC-Myc modules in gastric cancers in *KC-OSKM* chimeric mice given Dox for longer than 1 week. Gastric tumor 1 was induced by Dox treatment for 1 week, which is followed by Dox withdrawal for 3 weeks. Gastric tumors 2 and 3 were obtained by 2 weeks-treatment followed by 2 weeks-withdrawal.

(B) Macroscopic image of gastric cancer in *C-OSKM* chimeric mice given Dox for 2 weeks followed by Dox withdrawal for 2 weeks.

(C) Representative histological images and immunostaining for Ki67, Sall4, and Afp in gastric cancers in *C-OSKM* chimeric mice.

(D) Activation of ESC-Core and ESC-Myc modules in human gastric cancers expressing *LIN28B* and *ERBB2*. Note that *LIN28B*-expressing gastric cancers, but not *ERBB2*-expressing gastric cancers, exhibit partial activation of ESC-Core or ESC-Myc module.

**Supplementary Figure 9**

Fig1D: Southern blotting for *Kras* and *p53* allele

*Kras* allele

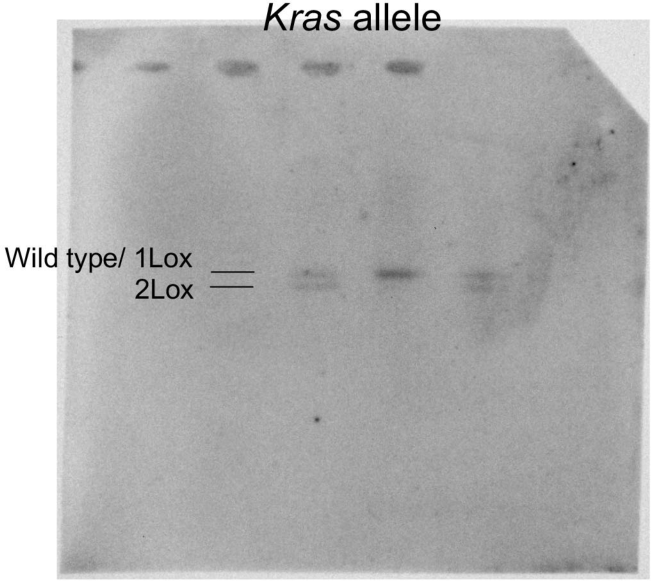


*p53* allele

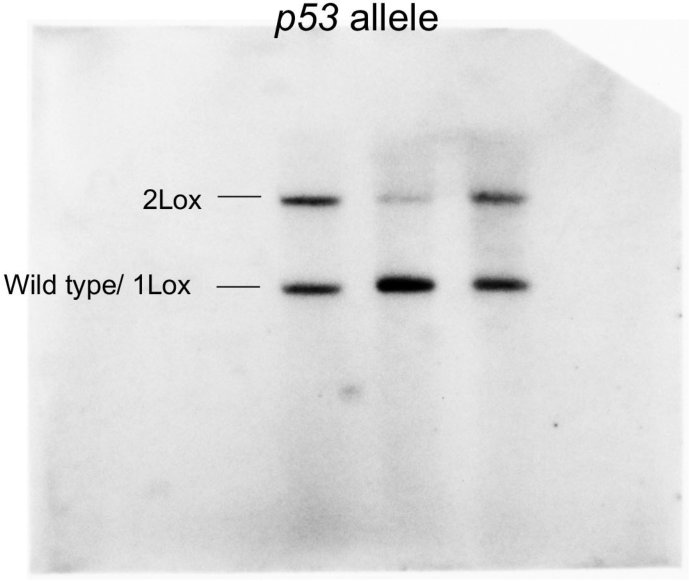


Fig 4H: Western blotting for ERK, pERK and EGFR antibody

ERK1/2

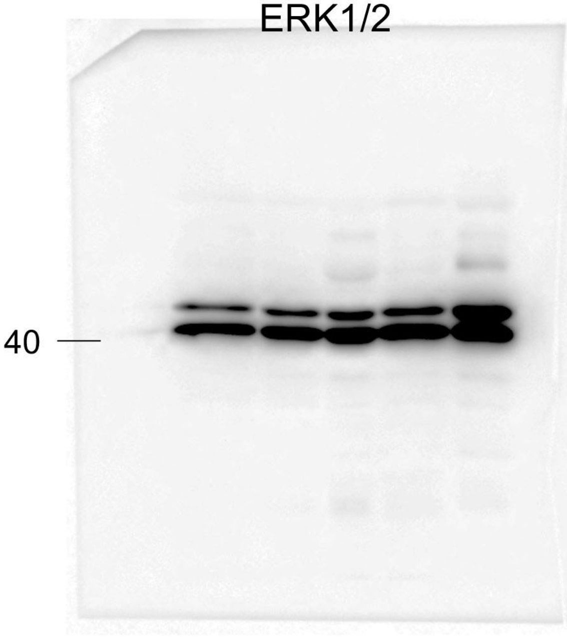


pERK1/2

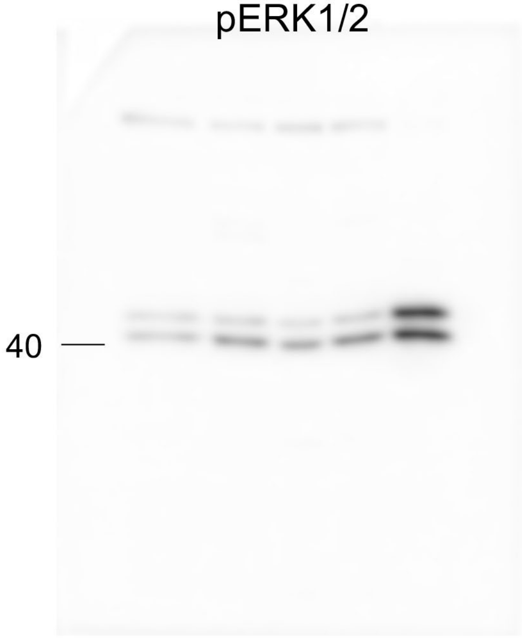


EGFR

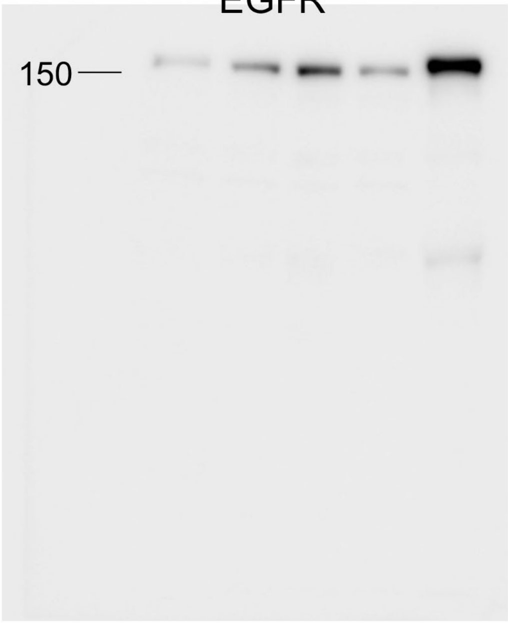

**Supplementary Figure 9: Uncropped Southern and Western blots**

## Gastric cancer development after prolonged expression of reprogramming factors

| Tissue No. | Genotype | Dox treatment | Chimerism | Tumor |
|---|---|---|---|---|
| 136 | ***C-OSKM*** | 2w/2wOFF | 40% | - |
| 138 | ***C-OSKM*** | 2w/2wOFF | 30% | + |
| 139 | ***C-OSKM*** | 2w/2wOFF | 30% | - |
| 140 | ***C-OSKM*** | 2w/2wOFF | 30% | - |
| 86 | ***C-OSKM*** | 1w/1wOFF | 40% | + |
| 87 | ***C-OSKM*** | 1w/1wOFF | 50% | - |
| 88 | ***C-OSKM*** | 1w/1wOFF | 40% | - |
| 91 | ***C-OSKM*** | 1w/1wOFF | 40% | - |
| 92 | ***C-OSKM*** | 1w/1wOFF | 70% | + |
| 28 | ***KC-OSKM*** | 1w/1wOFF | 20% | + |
| 29 | ***KC-OSKM*** | 1w/1wOFF | 50% | + |
| 34 | ***KC-OSKM*** | 1w/2wOFF | 50% | + |
| 35 | ***KC-OSKM*** | 2w/1wOFF | 50% | + |
| 37 | ***KC-OSKM*** | 2w/2wOFF | 60% | + |
| 40 | ***KC-OSKM*** | 2w/19daysOFF | 60% | + |
| 45 | ***KC-OSKM*** | 2w/2wOFF | 30% | + |
| 50 | ***KC-OSKM*** | 2w/2wOFF | 50% | Teratoma |
| 82 | ***KC-OSKM*** | 1w/1wOFF | 20% | + |
| 83 | ***KC-OSKM*** | 1w/1wOFF | 50% | + |
| 84 | ***KC-OSKM*** | 1w/1wOFF | 70% | + |
| 85 | ***KC-OSKM*** | 1w/1wOFF | 40% | - |
| 134 | ***KC-OSKM*** | 2w/2wOFF | 30% | + |
| 135 | ***KC-OSKM*** | 2w/2wOFF | 30% | + |
| 193 | ***KC-OSKM*** | 2w/1wOFF | 20% | + |
| 195 | ***KC-OSKM*** | 2w/3daysOFF | 40% | + |
| 210 | ***KC-OSKM*** | 2w/2wOFF | 40% | + |
| 224-1 | ***KC-OSKM*** | 2w/2wOFF | 20% | + |
| 224-2 | ***KC-OSKM*** | 2w/2wOFF | 20% | - |
| 225-1 | ***KC-OSKM*** | 2w/2wOFF | 40% | + |
| 225-2 | ***KC-OSKM*** | 2w/2wOFF | 40% | + |
| 226-1 | ***KC-OSKM*** | 2w/2wOFF | 50% | Teratoma |
| 226-2 | ***KC-OSKM*** | 2w/2wOFF | 40% | - |

**Expression of pluripotency-related proteins in human AFP-producing cancers**

| | SALL4 | LIN28A | LIN28B |
|---|---|---|---|
| No. 1 | + | + | - |
| No. 2 | - | - | - |
| No. 3 | + | + | + |
| No. 4 | + | - | + |
| No. 5 | + | + | + |
| No. 6 | + | + | + |
| No. 7 | - | - | - |
| No. 8 | + | + | + |
| No. 9 | - | - | - |
| No. 10 | - | - | + |
| No. 11 | - | - | + |
| No. 12 | + | + | + |
| No. 13 | + | - | + |
| No. 14 | + | - | + |
| No. 15 | - | - | + |
| No. 16 | - | - | - |
| No. 17 | ± | - | - |
| No. 18 | ± | - | + |
| No. 19 | + | ± | + |
| No. 20 | + | - | - |
| No. 21 | + | - | + |
| No. 22 | + | + | + |
| No. 23 | - | - | - |
| No. 24 | + | - | ± |
| No. 25 | + | + | + |

**Supplementary Table 3: Cloning primers**

| | | |
|---|---|---|
| *Sall4* | Fw | ATGTCGAGGCGCAAGCAG |
| | Rv | TTAGCTGACAGCAATCTTATTTTCCTC |
| *Nanog* | Fw | ATGAGTGTGGGTCTTCCTGGTC |
| | Rv | TCATATTTCACCTGGTGGAGTCACA |
| *Esrrb* | Fw | ATGGACGTGAGCGAACTGTG |
| | Rv | TTAGACTTTAGCTTCGAGCATTTCGAGG |
| *Lin28a* | Fw | ATGGGCTCGGTGTCCAACC |
| | Rv | TCAATTCTGGGCTTCTGGGAGC |
| *Mist1* | Fw | ATGAAGACCAAAAACCGGCCC |
| | Rv | CTAGCTCCCCTCTCTGAAGCTG |
| *mCherry* | Fw | GCCACCATGGTGAGCAAGG |
| | Rv | TTACTTGTACAGCTCGTCCATGCC |

**Supplementary Table 4: Primers for probes in Southern bloting**

| | | |
|---|---|---|
| *Pdx1* allele | Fw | TCAAGCCCTGGTTTTGTTTT |
| | Rv | CAGGAAGAGGCGTATCCAAA |
| *Kras* allele | Fw | AACTGCATTTTCCCCTCTTG |
| | Rv | AACTATCTGTGGGAAATGGGTTT |
| *p53* allele | Fw | GGAAAACTGCTGTCCTCGAC |
| | Rv | TTACAGCTGAAGCCACGAAA |

Supplementary Table 5: Genotyping Primers

| | | |
|---|---|---|
| *LSL-Kras G12D* | Fw | CTAGCCACCATGGCTTGAGT |
| | Rv | TCCGAATTCAGTGACTACAGATG |
| *LSL-p53 R172H* | Fw | AGCTAGCCACCATGGCTTGAGTAAGTCTGCA |
| | Rv | CTTGGAGACATAGCCACACTG |
| *Rosa LSL-rtTA3* | Fw | CTGGGAGTTGAGCAGCCTAC |
| | Rv | CGATGTGAGAGGAGAGCACA |
| *Rosa LSL-LacZ* | Fw | TTCACTGGCCGTCGTTTTACAACGTCGTGA |
| | Rv | ATGTGAGCGAGTAACAACCCGTCGGATTCT |
| *Rosa allele* | Fw | GCGAAGAGTTTGTCCTCAACC |
| | Rv1 | AAAGTCGCTCTGAGTTGTTAT |
| | Rv2 | GGAGCGGGAGAAATGGATATG |
| *Col1a1 allele* | Fw | CCCTCCATGTGTGACCAAGG |
| | Rv1 | GCACAGCATTGCGGACATGC |
| | Rv2 | GCAGAAGCGCGGCCGTCTGG |
| *Pdx1 IRES-Cre* | Fw | AGAGCCGGAGCAAGATTGT |
| | Rv1 | TCAGAAGCTCAGGGCTGTTT |
| | Rv2 | AGGAACTGCTTCCTTCACGA |
| *Sry* | Fw | CGTGGTGAGAGGCACAAGTT |
| | Rv | AGGCAACTGCAGGCTGTAAA |

Supplementary Table 6: qPCR Primers

| | | |
|---|---|---|
| *Amy* | Fw | GGGAGGACTGCTATTGTCCA |
| | Rv | TGGTATCTTTCCCACCAAGG |
| *Mist1* | Fw | CAGCGGATGCATAAACTCAA |
| | Rv | GCGGCTGCTGGACATAGTA |
| *Ptf1a* | Fw | AACCAGGCCCAGAAGGTTAT |
| | Rv | CCTCTGGGGTCCACACTTTA |
| *CK19* | Fw | ACCCTCCCGAGATTACAACC |
| | Rv | CAAGGCGTGTTCTGTCTCAA |
| *Tff1* | Fw | ATCTGTGTCCTCGCTGTGGT |
| | Rv | TCAAAACAGCAACCTCTCTCC |
| *Muc5ac* | Fw | TCCCACCTCTGAAGACCATC |
| | Rv | CTCATAGGCATCCCCACAGT |
| *mCherry* | Fw | GAGATCAAGCAGAGGCTGAA |
| | Rv | TCGTACTGTTCCACGATGGTG |
| *Sall4* | Fw | GCCCCTCAACTGTCTCTCTG |
| | Rv | GGGAGCTGTTTTCTCGACTG |
| *Nanog* | Fw | AAGTACCTCAGCCTCCAGCA |
| | Rv | GTGCTGAGCCCTTCTGAATC |
| *Esrrb* | Fw | CAAGGAGGGAGTGAGACTGG |
| | Rv | GGAGGCATGGCATACAGTTT |
| *Lin28a* | Fw | GTCTTTGTGCACCAGAGCAA |
| | Rv | CTTTGGATCTTCGCTTCTGC |

Supplementary Table 7: Immunostaining Antibodies

| Primary Antibodies | SOURCE | Catalog Number | Dilution |
|---|---|---|---|
| Goat polyclonal anti-AFP | Santa Cruz | Cat#sc-8108 | ×300 |
| Rabbit polyclonal anti-Pancreatic Amylase | Abcam | Cat#ab21156 | ×400 |
| Rabbit monoclonal anti-HA-Tag | Cell Signaling | Cat#3724 | ×50 |
| Rabbit monoclonal anti-Lin28a | Cell Signaling | Cat#8706 | ×500 |
| Rabbit polyclonal anti-Lin28b | Cell Signaling | Cat#4196 | ×100 |
| Rabbit monoclonal anti-Phospho-p44/42 Erk1/2 | Cell Signaling | Cat#4376 | ×400 |
| Rabbit monoclonal anti-Nanog | Cell Signaling | Cat#8822 | ×200 |
| Mouse monoclonal anti-Oct3/4 | BD Transduction Labolatories | Cat#611203 | ×400 |
| Rabbit monoclonal anti-Ki67 | Abcam | Cat#ab16667 | ×200 |
| Rat monoclonal anti-CK19 | DSHB | Cat#TROMA-Ⅲ | ×500 |
| Mouse monoclonal anti-Sall4 | Abnova | Cat#H00057167-M03 | ×500 |
| Mouse monoclonal anti-Esrrb | PPMX | Cat#PP-H6705-00 | ×500 |
| Rabbit monoclonal anti-GFP | Abcam | Cat#ab183734 | ×200 |
| Mouse monoclonal anti-CDKN2A/p16 INK4a | Abcam | Cat#ab54210 | ×500 |
| Mouse monoclonal anti-p21 | Santa Cruz | Cat#sc-6246 | ×500 |
| Rabbit polyclonal anti-p53 | Leica Biosystems | Cat#NCL-L-p53-CM5p | ×500 |
| Rabbit monoclonal anti-pRB | Cell Signaling | Cat#8516 | ×500 |
| Rat monoclonal anti-RFP | chromotek | Cat#5F8 | ×500 |
| Mouse monoclonal anti-phospho H2AX | Millipore | Cat#05-636 | ×500 |
| Rabbit monoclonal anti-EGFR | abcam | Cat#ab52894 | ×500 |
| **Secondary Antibodies, etc** | **SOURCE** | **Catalog Number** | **Dilution** |
| New Hematoxylin Solution for HE staining | Muto Pure Chemicals | Cat#30141 | ×1 |
| New Eosin Solution | Muto Pure Chemicals | Cat#3208-1 | ×1 |
| Hystofine Simplestain MAX-PO mouse | Nichirei Bioscience | Cat#414321 | ×1 |
| Hystofine Simplestain MAX-PO rabbit | Nichirei Bioscience | Cat#414341 | ×1 |
| Hystofine Simplestain MAX-PO goat | Nichirei Bioscience | Cat#414351 | ×1 |
| Hystofine Simplestain MAX-PO rat | Nichirei Bioscience | Cat#414311 | ×1 |
| DAPI | Invitrogen | Cat#D21490 | ×500 |
| CF488A Donkey Anti-Rat IgG | Biotium | Cat#20027 | ×150 |
| CF555 Donkey Anti-Rabbit IgG | Biotium | Cat#20038 | ×150 |

Western Blotting antibodies

| Primary Antibodies | SOURCE | Catalog Number | Dilution |
|---|---|---|---|
| Rabbit monoclonal anti-EGFR | abcam | Cat#ab52894 | ×1000 |
| Mouse monoclonal anti-p44/42 ERK1/2 | Cell Signaling | Cat#4696 | ×2000 |
| Rabbit monoclonal anti-Phospho-p44/42 Erk1/2 | Cell Signaling | Cat#4376 | ×1000 |
| Rabbit monoclonal anti-AKT | Cell Signaling | Cat#4691 | ×1000 |
| Rabbit monoclonal anti-phospho-AKT | Cell Signaling | Cat#4060 | ×2000 |
| **Secondary Antibodies** | **SOURCE** | **Catalog Number** | **Dilution** |
| Anti-rabbit IgG, HRP linked whole antibody from Donkey | GE Healthcare | Cat#NA934 | ×5000 |
| Anti-mouse IgG, HRP linked whole antibody from Donkey | GE Healthcare | Cat#NA931 | ×5000 |

Antibodies for Chromatin Immunoprecipitation Sequence

| Antibodies | SOURCE | Catalog Number |
|---|---|---|
| Mouse monoclonal anti-acetyl Histone H3 (Lys27) | Monoclonal Antibody Lab | 308-34843 |
| Mouse IgG2a Isotype Control-ChIP Grade | Abcam | ab18413 |
